# Percutaneous Management of Acquired Right Ventricular Outflow Tract Obstruction due to Giant Coronary Vein Graft Aneurysm

**DOI:** 10.1080/24748706.2018.1535206

**Published:** 2018-10-29

**Authors:** Matthew J. Daniels, James D. Newton, Andrew D. Kelion, Mario Petrou, Oliver J. Ormerod

**Affiliations:** a Division of Cardiovascular Medicine, Radcliffe Department of Medicine, University of Oxford, Oxford, UK; b BHF Centre of Research Excellence, University of Oxford, Oxford, UK; c Directorate of Cardiovascular Medicine and Surgery, Oxford University NHS Hospitals Trust, Oxford, UK

**Figure 1.**
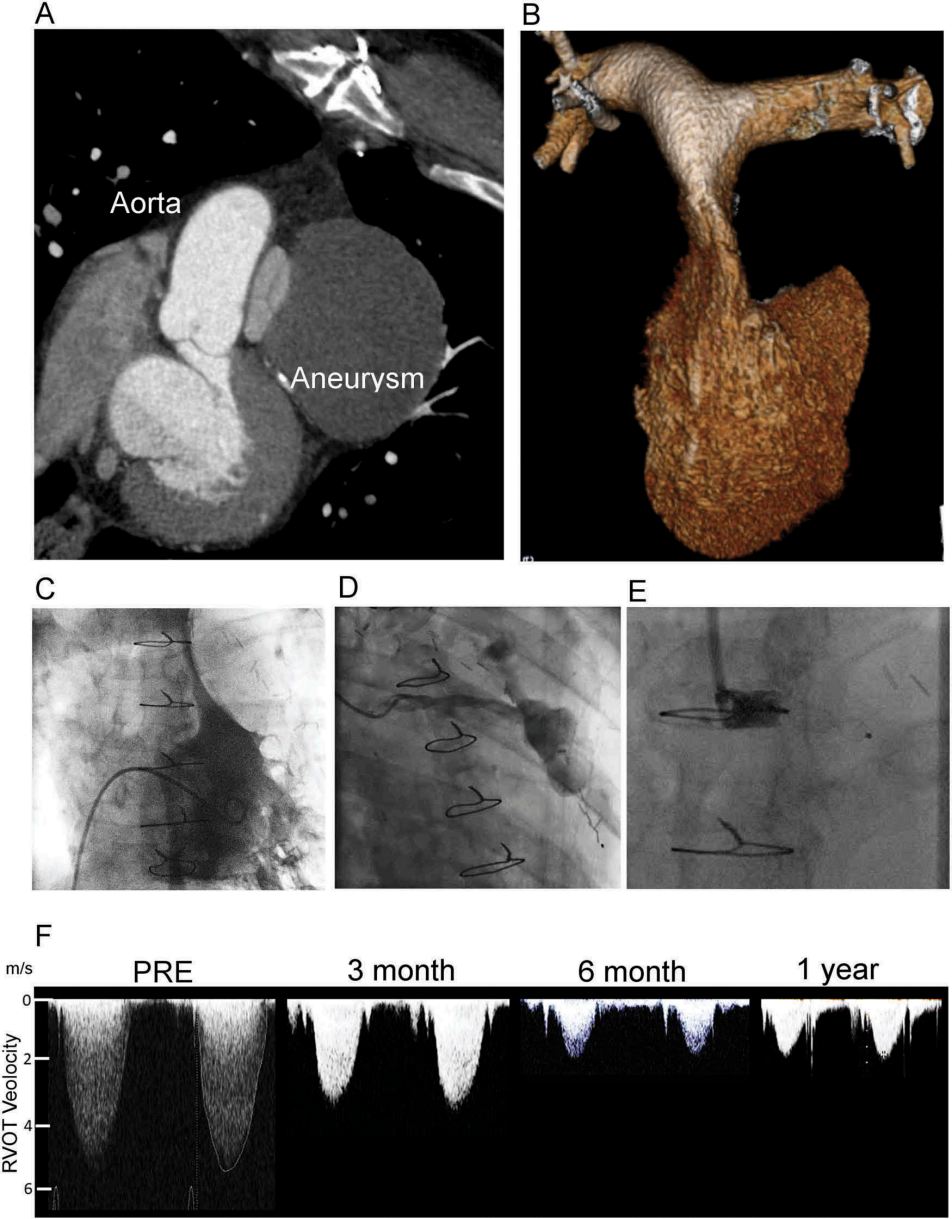
Acquired right ventricular outflow tract (RVOT) obstruction due to coronary graft aneurysm treated by percutaneous graft occlusion. (A) 2D multidetector computed tomography (MDCT) showing compression of the pulmonary valve between the graft aneurysm and the native aorta. (B) 3D MDCT reconstruction of the RV and pulmonary arteries showing the slit-like appearance of the main pulmonary artery. (C) Right ventriculography shows a minimum dimension of 2.2 mm, with an invasive pressure of 83 mmHg. (D) Graft angiography demonstrates the large aneurysm and feeding vessel. (E) An 8-mm AVPII device was used to occlude the graft with the expectation that thrombus would form and ultimately resorb. (F) Serial non-invasive Doppler assessments of the RVOT velocity show progressive and then stable reduction from 5.1 m/s at presentation to 1.7 m/s in follow up. RV dimensions, and hypertrophy normalize over this period. The patient was well and able to return to work at 6 months.

A 56-year-old fireman previously revascularized by coronary artery bypass presented unable to work 9 years following index surgery. Investigations revealed extrinsic compression of the right ventricular outflow tract between a coronary vein graft aneurysm and the ascending aorta (Figure 1A,B). Invasive systolic right ventricular pressure was elevated (83 mmHg peak pressure), with dynamic outflow tract obstruction (Figure 1C, Supplemental Video 1). Following heart team discussion in the absence of distal run off from the graft aneurysm the patient was offered transcatheter closure of the feeding vessel. The vein graft was cannulated with a JR4 catheter, sized (Figure 1D), and an 0.035ʹʹ Terumo wire used to advance distally to position an Amplatzer superstiff wire in the aneurysm. A 5F Amplatzer Torquevue 2 delivery system was used to deploy an 8 mm AVPII device within the proximal part of the vein graft. Non-invasive follow-up over the next 12 months showed resolution of acquired RVOT obstruction. The patient returned to work at 6 months

Stenosis of a tube is caused by internal, intramural, or external factors. Compression of the main pulmonary artery has previously been reported due to giant aneurysms of the native coronary vessels, pathologies of the aortic root (unruptured sinus of val salva aneurysm,^1^ and dissection), and mediastinal masses. Isolated case reports of acquired obstruction post-congenital^2^ or adult^3^ cardiac surgery involving the great vessels are also known but this case adds to the list of cardiac structures that may be compressed by coronary graft aneurysm, percutaneous treatment of which appears to be uncomplicated and effective.
